# Medical education at the crossroads. Part II: postgraduate training and the future of clinical expertise

**DOI:** 10.1177/01410768251395405

**Published:** 2025-12-01

**Authors:** Louella Vaughan, Martin McKee

**Affiliations:** 1 Barts Health NHS Trust, London E1 1BB, United Kingdom; 2 Department of Internal Medicine, Amsterdam University Medical Centre, Amsterdam 1081 HV, The Netherlands; 3 London School of Hygiene & Tropical Medicine, London WC1H 9SH, United Kingdom

## Introduction

NHS England’s 10-Year Plan, *Fit for the Future* (2025), outlines an ambitious vision for transforming healthcare delivery and workforce development.^1^ The Neighbourhood Health Service is at its centre, prioritising decentralised, digitally enabled and prevention-focused care. These changes are accompanied by sweeping reforms to postgraduate medical education (PGME), now reimagined as a flexible, service-responsive continuum of lifelong learning.

The Plan departs from traditional time-bound training and formal examinations, favouring modular progression, competency-based assessment and digital platforms. It also introduces new roles for alternative providers and decentralised regulation, challenging the authority of established institutions such as the Deaneries, Royal Colleges and the General Medical Council (GMC). These changes reflect broader ideological shifts and raise critical questions about educational coherence, professional standards and the future of clinical expertise.

This commentary examines the implications of the Plan for PGME and should be read in conjunction with the accompanying one on undergraduate medical education, which describes how both were developed using the ‘red teaming’ concept.^2^ While the Plan offers opportunities for innovation and adaptability, it also risks fragmenting PGME, weakening the foundations of clinical practice and public trust.

## Restructuring postgraduate training

The NHS England 10-Year Plan proposes a fundamental reconfiguration of PGME, dissolving long-standing boundaries between formal training and continuing professional development. Following the 1995 Calman reforms,^3^ PGME has been defined as a structured phase of education, culminating in certification and entry into independent practice. Continuing professional development, by contrast, has focused on professional education beyond initial qualification. The Plan collapses this distinction, promoting a seamless continuum of skill acquisition under the banner of ‘lifelong learning’. While this may enhance flexibility, it risks undermining the coherence of educational pathways and the clarity of professional milestones.

Central to this restructuring is the shift to modular progression. The Plan expands the use of the Certificate of Eligibility for Specialist Registration (CESR), initially designed for overseas-trained doctors. Under this model, doctors may accumulate competencies through varied experiences, potentially outside formal training programmes, and submit evidence for recognition. The traditional Certificate of Completion of Training (CCT) will no longer be the default pathway. While this may address workforce shortages, it risks fragmenting the training experience, weakening peer networks and eroding standardisation.

The Plan also signals a reduced emphasis on formal examinations, particularly those administered by the Medical Royal Colleges. Instead, it promotes competency-based assessments, real-time feedback and continuous skill development, arguing that ‘train[ing] ‘to role’ – often requiring individuals to complete years of training’ is unnecessary as there are ‘significant opportunities to move beyond traditional professional boundaries in a safe and productive way’. While this may reduce stress and improve responsiveness, it raises concerns about rigour, fairness and consistency. Without standardised benchmarks, it becomes difficult to ensure that all trainees meet the same level of competence, regardless of location or supervisor.

Together, these changes reflect a broader ideological shift toward service responsiveness and individualised progression. However, they also risk fragmenting PGME into a patchwork of experiences, lacking the depth, structure and accountability that have historically defined medical training. The erosion of standardised pathways and assessments may compromise the quality of education, the integrity of professional certification and ultimately, patient safety. Safeguards will be needed to ensure that flexibility does not come at the expense of rigour and public trust.

## Delivery and supervision of education

The 10-Year Plan proposes a major overhaul of how postgraduate medical education is delivered and supervised, centred on the introduction of a ‘digitally led platform for education, skills and training’ for all NHS staff. This platform is designed to support modular, competency-based learning and real-time feedback, replacing traditional models of structured progression and formal assessment. Crucially, the Plan invites alternative providers, including commercial and international organisations, to contribute content, challenging the long-standing dominance of Deaneries and Royal Colleges in postgraduate education.

The Plan posits that PGME should be driven by local service needs, emphasising ‘training to task’ rather than ‘training to role’, with ‘real-time feedback and continuous skill acquisition’ embedded with service delivery. This has been described as a ‘cheesecake factory’ model,^4^ touted as a means to make care more efficient and consistent, but leading to supervision becoming a mechanism for ensuring protocol adherence and task completion, rather than a dynamic process of mentorship and clinical reasoning. This risks eroding epistemic depth, the deep, reflective and context-sensitive knowledge that has produced well-rounded medical professionals capable of complex clinical reasoning and able to occupy senior roles in the health service. While this break with a system of national standards might enhance flexibility and innovation, it raises serious concerns about consistency, educational depth and future leadership of the NHS.

The current model of PGME relies on structured supervision, reflective practice and competency-based assessments overseen by experienced clinicians. The proposed system increases the burden on supervisors, who may have to work without the support of standardised benchmarks or rotational training frameworks. This likely will increase variability in supervision quality and risks undermining the reliability of PGME. It also risks fragmenting supervision, detaching it from its pedagogical roots, and opens trainees to the possibility of inconsistent or inequitable evaluations. Real-time feedback embedded within service delivery may prioritise operational efficiency over educational value, reducing opportunities for mentorship, critical reflection and longitudinal development.

The tension between service delivery and pedagogical integrity is particularly acute. As education becomes increasingly embedded in frontline care, there is a risk that training will be subordinated to workforce demands. Trainees may be viewed more as service providers than learners, with supervision focused on task completion rather than professional growth.

In sum, while the Plan’s proposals offer potential efficiencies, they risk weakening the foundations of postgraduate education. Safeguards are needed to ensure that flexibility does not come at the expense of rigour, consistency and the formative experiences that underpin clinical expertise and professional identity.

## Regulatory implications

The proposals in the Plan to prioritise local service needs and decentralise oversight also have significant implications for regulation. Shifts from the traditional model of national curricula, structured progression and uniform expectations across institutions to an employer-led model threaten to erode shared standards and benchmarks. The Plan hints heavily at the removal of formal examinations, considered to be constraints to service delivery. Without clear criteria or oversight, assessments are likely to become subjective, variable and overly influenced by local service pressures. This undermines the reliability of training outcomes and may compromise the quality of clinical education across regions, as well as patient safety.

Traditional regulatory bodies such as the Deaneries, Royal Colleges and the GMC may be weakened under this model. The GMC’s proposed shift to task-specific registration further complicates oversight, especially in the absence of robust governance structures. As national standards are dismantled and replaced by local discretion, a regulatory vacuum may emerge, one in which accountability is diffuse, quality assurance is inconsistent and both trainees and supervisors are exposed to greater risk.

These proposals are closely tied to the Plan’s ‘skills escalator’ model, which assumes that individuals will continuously acquire new competencies and progress autonomously through the system. Entry thresholds into professional education are relaxed, allowing individuals to begin training without traditional academic qualifications and advance to senior clinical roles. While this may improve accessibility and support workforce expansion, it sets up competing pathways, with medical residents jostling with non-medical professionals for educational opportunities. It also raises concerns about the foundational preparedness of different health workers, illustrated by what the Plan concedes were problems with the introduction of physician assistants.^5^ That unfortunate experience raised questions about the ability of some groups to engage with complex clinical material.^6^

While the Plan’s regulatory reforms aim to enhance flexibility and responsiveness, they risk destabilising the foundations of postgraduate medical education. Safeguards must be introduced to ensure that decentralisation does not erode professional standards, compromise patient safety or weaken public trust in the medical profession.

## Career progression and professional identity

The 10-Year Plan introduces multiple pathways to consultancy, notably expanding the CESR as an alternative to the traditional CCT. While this modular, experience-based route may improve flexibility and address workforce shortages, it risks creating informal hierarchies within the profession. Consultants who progress via CESR may be perceived as less rigorously trained than those who follow conventional pathways, potentially affecting career prospects, institutional prestige and patient confidence.

This fragmentation also threatens the international recognition of UK medical qualifications. The erosion of standardised training and formal examinations may lead other jurisdictions to question the equivalence of British postgraduate education, reducing the ability of British-trained doctors, once highly sought after, to obtain full registration and employment in other jurisdictions. Of course, this may be the intention.

The Plan further redefines the doctor’s role, positioning senior clinicians primarily as supervisors of multidisciplinary teams rather than direct providers of care. This managerial shift risks diminishing the epistemic depth traditionally associated with medical expertise, replacing reflective clinical reasoning with adherence to protocols and oversight of tasks. The loss of mentorship and bedside teaching weakens the formative relationships that have long shaped professional identity.

The desire for the NHS workforce to ‘move beyond traditional professional boundaries’ is coupled with the expansion of multidisciplinary teams, which will be redefined to include non-traditional groups such as those working in housing, employment and care as part of the new Neighbourhood Health Service. The potential impact should not be underestimated. The proposals risk reducing professional collaboration to a series of interchangeable tasks rather than a synthesis of complementary expertise. If all professions are trained to think in the same way, guided by protocols and stripped of disciplinary nuance, the richness of interdisciplinary dialogue may be lost. This not only affects the quality of care but also the integrity of professional formation across the health system.

Symbolic belonging, such as membership in Royal Colleges, is also at risk. As training becomes more fragmented and commercialised, the institutional anchors that foster professional community and continuity may be devalued. This erosion of identity is compounded by the flattening of professional boundaries within multidisciplinary teams. While collaboration is essential, the homogenisation of roles and training threatens to dilute the distinct contributions of each profession.

The Plan’s reforms risk destabilising the coherence of medical careers and the integrity of professional identity. Safeguards are needed to preserve depth, mentorship and the symbolic structures that support clinical excellence and public trust.

## Conclusion

The NHS England 10-Year Plan presents both significant opportunities and profound risks for postgraduate medical education. While its emphasis on flexibility, digital innovation and service responsiveness may enhance adaptability, it also threatens to fragment training, dilute standards and destabilise professional identity. The erosion of central oversight and structured progression demands urgent attention. Reform must be guided by clarity, coherence and robust safeguards to ensure educational rigour and public trust. A balanced approach is essential, one that embraces innovation without compromising the integrity of postgraduate medical education or the quality of clinical care.

## Declarations

**Ethical approval:** This article does not contain any studies with human or animal participants.

**Guarantor:** LV.

**Consent to participate:** Not applicable.

**Consent for publication:** Not applicable.

**Provenance:** Not commissioned; editorial review.
